# Dracunculin Inhibits Adipogenesis in Human Bone Marrow-Derived Mesenchymal Stromal Cells by Activating AMPK and Wnt/β-Catenin Signaling

**DOI:** 10.3390/ijms23020653

**Published:** 2022-01-07

**Authors:** Fatih Karadeniz, Jung Hwan Oh, Hyun Jin Jo, Jiho Yang, Hyunjung Lee, Youngwan Seo, Chang-Suk Kong

**Affiliations:** 1 Marine Biotechnology Center for Pharmaceuticals and Foods, College of Medical and Life Sciences, Silla University, Busan 46958, Korea; karadenizf@outlook.com (F.K.); wjdghks0171@naver.com (J.H.O.); 2 Department of Food and Nutrition, College of Medical and Life Sciences, Silla University, Busan 46958, Korea; genie970909@naver.com (H.J.J.); fwlgh97@naver.com (J.Y.); ppbbaa04021@naver.com (H.L.); 3 Division of Marine Bioscience, Korea Maritime and Ocean University, Busan 49112, Korea; ywseo@kmou.ac.kr

**Keywords:** adipogenesis, AMPK, dracunculin, hBM-MSC, Wnt/β-catenin

## Abstract

Increased bone marrow adiposity is widely observed in patients with obesity and osteoporosis and reported to have deleterious effects on bone formation. Dracunculin (DCC) is a coumarin isolated from *Artemisia* spp. but, until now, has not been studied for its bioactive potential except antitrypanosomal activity. In this context, current study has reported the anti-adipogenic effect of DCC in human bone marrow-derived mesenchymal stromal cells (hBM-MSCs). DCC dose-dependently inhibited the lipid accumulation and expression of adipogenic transcription factors peroxisome proliferator-activated receptor γ (PPARγ) and CCAAT/enhancer binding protein α (C/EBPα) in hBM-MSCs induced to undergo adipogenesis. To elucidate its action mechanism, the effect of DCC on Wnt/β-catenin and AMPK pathways was examined. Results showed that DCC treatment activated Wnt/β-catenin signaling pathway via AMPK evidenced by increased levels of AMPK phosphorylation and Wnt10b expression after DCC treatment. In addition, DCC treated adipo-induced hBM-MSCs exhibited significantly increased nuclear levels of β-catenin compared with diminished nuclear PPARγ levels. In conclusion, DCC was shown to be able to hinder adipogenesis by activating the β-catenin via AMPK, providing potential utilization of DCC as a nutraceutical against bone marrow adiposity.

## 1. Introduction

Worldwide prevalence of obesity is considered to be one of the main promoters for several common metabolic diseases such as diabetes, cardiovascular problems, and cancer [1]. Reported links between obesity progression and the prevalence of various other diseases have become a big concern for public health. Obesity is characterized basically with inflammation caused by the hormonal secretions of excessive adipose tissue formation and consequent lipid accumulation in the body. Formation of the adipose tissue and accumulating lipid are results of overstimulated adipogenesis, which is an intricate differentiation process of pre-adipocytes involving several intertwined signaling pathways resulting in differentiation into mature adipocytes [2]. Maturation of adipocytes is characterized with intracellular lipid accumulation and adipocyte-specific gene expressions. Initiation of the adipogenesis is strictly regulated by adipogenic transcription factors of which peroxisome proliferator activated receptor gamma (PPARγ) and CCAAT/enhancer binding proteins (C/EBP) are the key factors [3]. Expression and transcriptional activities of these adipogenic factors, as well as their downstream effector proteins, are mandatory for the progression of adipogenesis.

In multipotent mesenchymal stromal cells (MSCs), increased adipocyte formation as a result of adipogenic differentiation promotes a set of bone disorders, osteoporosis being the most prevalent one [4,5]. Adipogenic tendencies of the bone marrow MSCs against osteogenic differentiation are the main causes of sponge-like bone formation seen in osteoporotic conditions. Several reports have suggested that wingless-type MMTV integration site (Wnt)/β-catenin is the positive regulator for the osteogenic differentiation of bone marrow MSCs, simultaneously suppressing the adipocyte differentiation [6]. In healthy individuals, negative regulation of adipogenesis via canonical Wnt/β-catenin pathway keeps the balance between adipocyte and osteocytes in bone structure. As the downstream effector of this canonical pathway, nuclear translocation of β-catenin negatively regulates the expression of PPARγ and C/EBPα, consequently inhibiting the adipogenesis [7]. Therefore, regulation of PPARγ and Wnt/β-catenin signaling might be considered as a target to prevent or control obesity via hindering adiposity.

Natural bioactive substances have been gaining increasing attention due to their ability to regulate adipogenic differentiation. To date, several types of phytochemicals have been reported to possess adipogenesis inhibitory properties [8]. Phenolic substances allocate a big portion of aforementioned bioactive phytochemicals. *Artemisia princeps*, also known as Korean or Japanese mugwort, is a plant native to Asia and is reported to possess adipogenesis inhibitory properties in mouse 3T3-L1 pre-adipocytes among its other in vitro bioactivities such as antioxidant, antimicrobial, anti-inflammatory and antitumor [9,10,11]. Several phenolic substances have been isolated from *A. princeps* as an active component. Eupatilin and jaceosidin flavonoids inhibited inflammation in mice [12] while 6-acetyl-2,2-dimethylchroman-4-one [13] and loliolide [14] inhibited the adipogenic differentiation of human bone marrow-derived mesenchymal stromal cells. Dracunculin, which is the simple name given to 6-methoxy-7,8-methylenedioxycoumarin, is a known coumarin that can be found mainly in mugworts. It has been isolated mostly from *Artemisia* species, tarragon (*Artemisia dracunculus*) being the first, which has given this compound its common name, dracunculin. Although several other coumarins were isolated from *Artemisia* species and their bioactivities have been reported, the literature lacks detailed information in terms of the health beneficial properties of dracunculin. Therefore, in this study, the potential anti-adipogenic effect of dracunculin (DCC) isolated from *A. princeps* has been evaluated. To the best of our knowledge, this is the first report that exhibits the effect of DCC on adipogenic differentiation.

## 2. Results

### 2.1. Effect of DCC on Lipid Accumulation of Adipo-Induced hBM-MSCs

**Figure 1:**
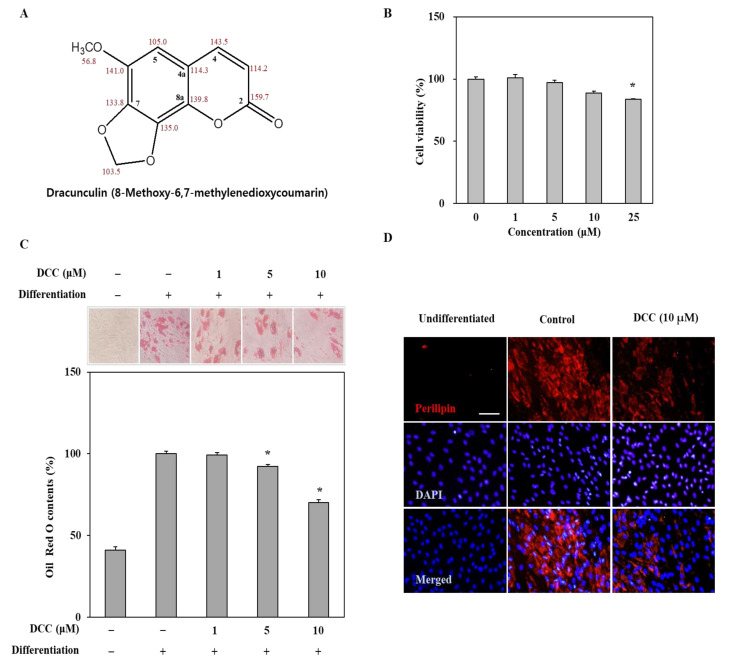
Effect of DCC on the lipid accumulation of adipo-induced hBM-MSCs. (**A**) The chemical structure of DCC. (**B**) Effect of DCC on the cell viability of hBM-MSCs. Viable cell amount was measured by quantification of MTT dye removed from cells after 3 days of DCC treatment. Cell viability was given as relative viable cell amount (%) of untreated control. (**C**) Effect of DCC on the lipid accumulation of adipo-induced hBM-MSCs on day 12. Intracellular lipid accumulation was measured by Oil Red O staining. (**D**) Effect of DCC on the expression of perilipin-1 in adipo-induced hBM-MSCs on day 12 analyzed by immunofluorescence staining. DAPI staining was used to highlight the nucleus of viable cells. Scale bar: 50 μm. * *p* < 0.05 vs. differentiated untreated control group.

The hBM-MSCs were treated with DCC (Figure 1A) at the concentrations of 1, 5, 10 and 25 μM for 3 days in order to evaluate their cytotoxicity. The cell viability was quantified by MTT assay after the 3-day treatment. The first statistically significant decrease in the viability of cells was observed after DCC treatment at a concentration of 25 μM (Figure 1B). The concentrations below that did not affect the cell viability. As a result, the following experiments were carried out using concentrations up to 10 μM.

Any effect of DCC on the lipid accumulation of adipo-induced cells was observed by Oil Red O staining. As shown in Figure 1C, DCC reduced the amount of lipid droplets in adipocytes on day 12 of differentiation in a dose-dependent manner compared to the untreated adipocytes that were only fed differentiation medium. The lipid droplet contents were quantified by the optical density values of retained stain in the adipocytes. At the most effective concentration (10 μM), DCC treatment significantly lowered the lipid droplet content by 29.88%. The effect of DCC on lipid droplets was also shown via the fluorescence staining of perilipin, the protein that coats the lipid droplets in adipocytes. DCC treatment at 10 μM resulted in significantly less perilipin visible in adipocytes compared to the untreated control group, as seen in Figure 1D.

### 2.2. DCC Suppresses the mRNA and Protein Expressions of Adipogenic Transcription Factors

**Figure 2:**
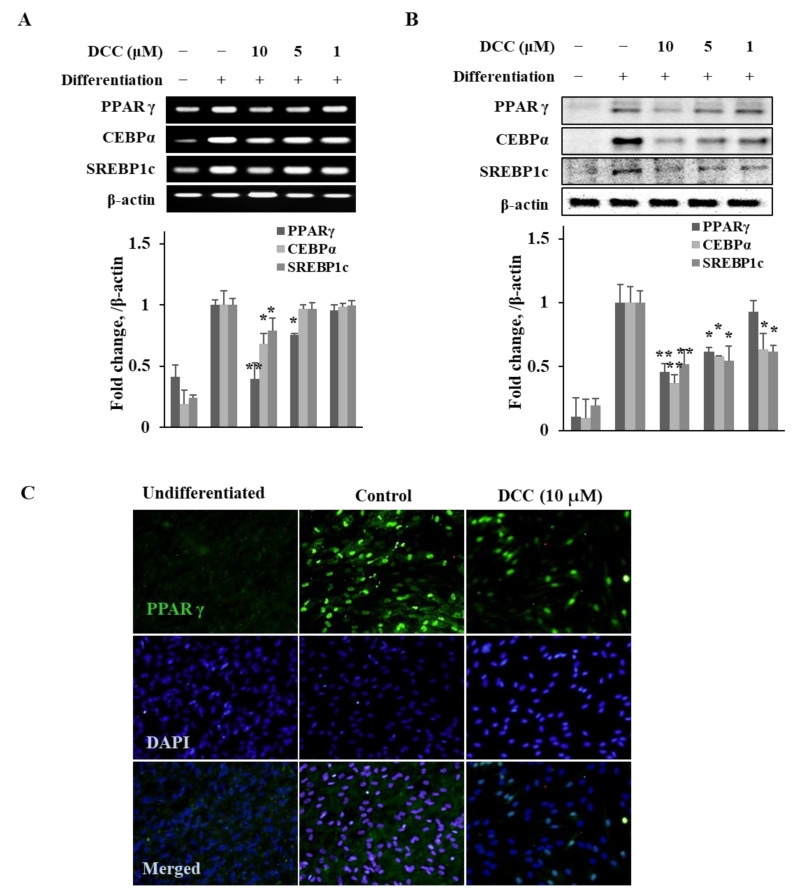
Effect of DCC on adipogenic transcription factor expressions. Analysis of mRNA (**A**) and protein (**B**) expressions in adipo-induced hBM-MSCs was carried out via RT-PCR and Western blot, respectively, on day 12 of differentiation. (**C**) Effect of DCC on the expression of perilipin-1 in adipo-induced hBM-MSCs on day 12, analyzed by immunofluorescence staining. DAPI staining was used to highlight the nucleus of viable cells. Scale bar: 50 μm. * *p* < 0.05, ** *p* < 0.01 vs. differentiated untreated control group.

Adipogenesis progresses via the activity of transcription factors such as PPARγ, C/EBP-α and SREBP1c [3]. Therefore, the effect of DCC on both the mRNA and protein expression levels of these adipogenesis-related transcription factors was determined. The hBM-MSCs were induced to differentiate along with varying concentrations of DCC (1, 5 and 10 μM,) and the mRNA and protein expressions of PPARγ, C/EBP-α and SREBP1c were analyzed by RT-PCR and Western blotting, respectively. The 3-day DCC treatment with the initial adipogenesis induction dose-dependently decreased the mRNA levels of tested transcription factors on day 12 of adipogenesis (Figure 2A). Similar results were obtained from Western blotting. Following DCC treatment, the protein levels of PPARγ, C/EBP-α and SREBP1c were all lowered in a dose-dependent manner (Figure 2B). In addition, the effect of DCC on the expression of PPARγ, the main regulator of adipogenesis, was evidenced by fluorescence staining. The adipocytes were stained with fluorescent antibody to highlight the PPARγ levels on day 12 of differentiation. Consistent with the previous results, PPARγ levels were significantly low in DCC (10 μM)-treated adipocytes compared with untreated control adipocytes (Figure 2C).

### 2.3. Effect of DCC on Wnt/β-Catenin, MAPK and AMPK Signaling Pathways

**Figure 3:**
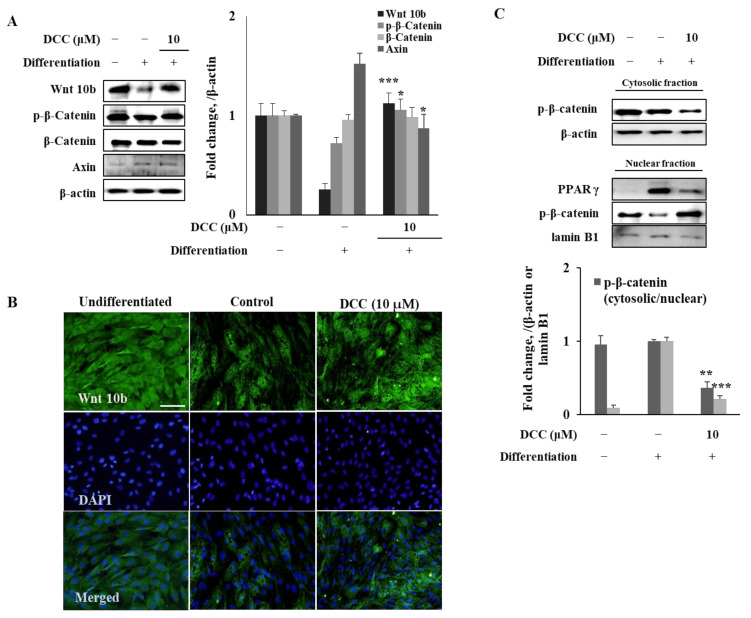
Effect of DCC on Wnt/β-catenin signaling pathway. Analysis of the protein expression levels of Wnt/β-catenin signaling pathway components (**A**,**C**) was carried out by Western blotting while Wnt10b expression levels were also observed via immunofluorescence staining (**B**) in adipo-induced hBM-MSCs on day 12. β-actin (for whole cell and cytosolic fraction) and lamin B1 (for nuclear fraction) were used as internal loading control. DAPI staining was used to highlight the nucleus of viable cells. Scale bar: 50 μm. * *p* < 0.05, ** *p* < 0.01, *** *p* < 0.001 vs. differentiated untreated control group.

To elucidate the mechanism behind the effect of DCC on the PPARγ-related adipogenic differentiation, other signaling cascades that were shown to be in crosstalk and differentially regulating adipogenesis were analyzed. Reports showed that canonical Wnt/β-catenin signaling has an opposing relation with PPARγ where activation of Wnt/β-catenin subsequently downregulates PPARγ signaling and, therefore, adipogenesis is suppressed [6]. In this context, the protein expression of Wnt/β-catenin signaling components were observed in adipo-induced hBM-MSCs treated with or without DCC. As seen in Figure 3A, DCC (10 μM) treatment increased Wnt10b protein levels, which were observed to be significantly downregulated in the adipo-induced untreated control group shown via fluorescence staining. The Western blotting results also showed similar results for Wnt10b protein levels (Figure 3B). However, the downstream effector and transcription factor of β-catenin levels were not affected by either adipogenesis or DCC treatment. This was also supported by the axin levels, which the DCC treatment could not decrease, as an indicator of activated Wnt/β-catenin pathway. On the other hand, DCC treatment slightly elevated Ser^552^ phosphorylated β-catenin levels. This phosphorylation of β-catenin via AMPK signaling stabilizes the transcription factor and consequently induces its nuclear translocation, which was previously noted as an adipogenesis suppressing mechanism [15]. Subcellular fractionation of proteins supported this hypothesis as seen in Figure 3C. Adipogenic differentiation resulted in a significant increase in nuclear PPARγ levels while the nuclear Ser^552^ phosphorylated β-catenin levels diminished. Presence of 10 μM DCC reverted these adipogenesis induced changes: decreasing nuclear presence of PPARγ transcription factor while stimulating the β-catenin nuclear translocation.

**Figure 4:**
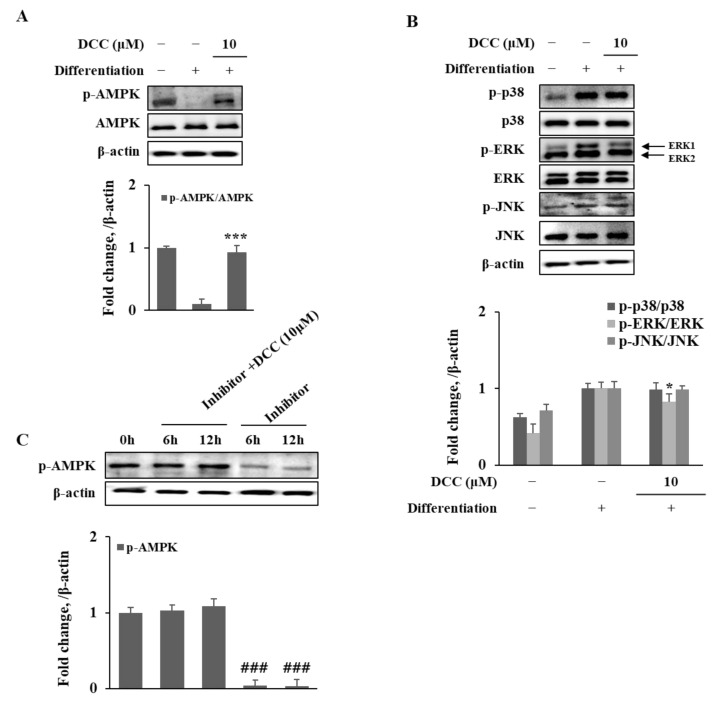
Effect of DCC on activation of AMPK (**A**) and MAPK (**B**) signaling pathways. Compound C was used as a negative control for the inhibition of AMPK activation (**C**). Analysis of protein expression was carried out by Western blotting of adipo-induced hBM-MSCs on day 12. β-actin was used as internal loading control. * *p* < 0.05, *** *p* < 0.001 vs. differentiated untreated control group. ### *p* < 0.001 vs. 0 h group.

To further support the reciprocal effect of DCC on the activation of Wnt/β-catenin and AMPK pathways, the activated AMPK protein levels were analyzed with or without the use of compound C, a known AMPK inhibitor. As shown in Figure 4A, adipo-induced hBM-MSCs exhibited a significant downregulation of AMPK activation, presented as totally diminished phosphorylated AMPK levels in comparison with the same AMPK levels. DCC (10 μM) treatment notably relieved the adipogenic suppression of AMPK activation and increased the phosphorylated AMPK levels while not affecting total AMPK level. Next, the effect of DCC on the MAPK activation was examined. Results showed that the phosphorylation of p38, JNK and ERK1/2 was significantly stimulated during adipogenesis (Figure 4B). However, DCC (10 μM) treatment was only able to decrease the ERK1/2 phosphorylation—it was not able to result in any significant change in p38 and JNK phosphorylation. The antagonistic relationship between AMPK and ERK1/2 might be the reason behind the DCC-mediated downregulation of ERK phosphorylation as the reports indicated that some anti-adipogenic phytochemicals act via AMPK-induced MAPK suppression [16,17].

Next, the effect of DCC on AMPK activation was investigated. The hBM-MSCs were treated with compound C in the presence or absence of DCC (10 μM). Only compound C-treated hBM-MSCs showed significantly decreased phosphorylated AMPK levels after 6 and 12 h of treatment (Figure 4C). Treating DCC along with compound C relieved the inhibitory effect of compound C on AMPK phosphorylation in a time-dependent manner. After 12 h of treatment, the DCC and compound C-treated group exhibited normal activated AMPK levels compared with the pre-treatment (0 h) hBM-MSCs.

## 3. Discussion

DCC is a coumarin derivative, also named as 7,8-Methylenedioxy-6-methoxycoumarin [18]. It has been known as a phytochemical for decades and is mainly isolated from *Artemisia* spp. Although *Artemisia* spp. are known to possess anti-adipogenic properties and DCC is a common phytochemical, the literature lacks studies on the bioactive potential of DCC. Like other coumarins similar to DCC in terms of structure, DCC is expected to possess some bioactive properties. To the best of our knowledge, the only bioactive property reported for DCC is its antitrypanosomal activity [19]. The current study proposes, for the first time, that DCC possesses bioactive potential to suppress adipogenic differentiation of bone marrow cells in vitro by activation of Wnt/β-catenin and AMPK pathways.

Increased adipogenic differentiation of bone marrow cells is suggested to be one of the causes for obesity-related bone complications such as osteoporosis [1]. Formation of adipocytes is characterized by accumulation of intracellular lipid droplets as a marker for mature adipocytes. In this study, DCC was shown to decrease the lipid accumulation of hBM-MSCs when they were induced to undergo adipogenesis compared with the untreated group that differentiated into mature adipocytes with substantial lipid accumulation. This result indicated that DCC treatment hindered adipogenesis at some point that the adipo-induced hBM-MSCs were not able to mature and accumulate lipid droplets.

The adipogenesis of mesenchymal stromal cells is regulated by a web of different signaling cascades and transcription factors [3]. The main adipogenic transcription factors that initiate and progress the adipocyte formation are PPARγ and C/EBP-α. PPARγ activation can stimulate the beginning of adipogenesis by itself via a positive feedback loop and crosstalk between C/EBP-α and its upstream activators, C/EBP-β and C/EBP-δ [20]. The adipogenic differentiation tendencies of hBM-MSCs, instead of osteoblastogenesis, are also regulated by this cascade very similarly to adipose tissue. Activation of this signaling cascade occurs at the early stages of adipogenic differentiation. Hence, in the current study, DCC was introduced to the cells during the initial induction of adipogenesis. The results showed that DCC inhibited the mRNA and protein levels of PPARγ and C/EBP-α, as well as adipocyte-specific SREBP1c, showing that DCC was able to suppress adipogenesis by interfering with the transcription factor signaling cascade.

Another known regulator of adipogenesis is Wnt/β-catenin signaling, which downregulates PPARγ and C/EBPα cascade when activated [6]. It has been shown that the activation of Wnt receptors negatively regulates the adipogenesis via suppression of the activity of PPARγ by nuclear translocation of β-catenin. Results showed that DCC treatment significantly increased the Wnt10b protein expression, which was suppressed in untreated adipocytes as an indicator of activated Wnt pathway. However, DCC was not able elevate the β-catenin protein levels as expected. This showed that DCC was able to activate the Wnt pathway in a non-canonical way. Therefore, Ser^552^ phosphorylated β-catenin levels were examined. When phosphorylated at Ser^552^ via AMPK, β-catenin is stabilized and accumulated in the nucleus, which consequently suppresses the PPARγ signaling [15]. DCC treatment significantly increased Ser^552^ phosphorylated β-catenin levels in adipo-induced hBM-MSCs but did not affect the total β-catenin levels, indicating that DCC was able to activate β-catenin transcriptional activity through AMPK. This was further supported by the nuclear levels of phosphorylated β-catenin and PPARγ. Adipocytes expressed a high amount of PPARγ compared with a minimal presence of phosphorylated β-catenin in the nucleus. However, DCC treatment reverted the nuclear protein profile of adipo-induced hBM-MSCs. DCC-treated cells showed increased levels of nuclear phosphorylated β-catenin and diminished PPARγ. This supported the suggestion that DCC hinders the adipogenesis via activated β-catenin signaling.

AMPK is also known as a negative regulator for adipogenesis via different ways, which include suppression of MAPK activation on ERK1/2 and activation of β-catenin via phosphorylation. The stimulation of AMPK activation by DCC treatment was shown in this study. The presence of DCC significantly increased the AMPK phosphorylation, which was suppressed during adipogenesis. In addition, DCC was able to revert the effects of AMPK inhibitor compound C, further suggesting strong AMPK activation activity for DCC. Abiola et al. [21] suggested a reciprocal crosstalk between AMPK and Wnt10b activation. They reported that activation of Wnt/beta-catenin signaling by means of elevated Wnt10b protein in skeletal muscle cells resulted in activated AMPK pathway and suppressed MAPK activation. These results are consistent with the findings of the current study, which explain the Wnt10b stimulation by DCC treatment along with AMPK-mediated non-canonical β-catenin activation. However, future studies are needed to determine the detailed mechanism of action for DCC to show where and how DCC interferes with the crosstalk between AMPK and Wnt/β-catenin signaling, which resulted in suppressed adipogenesis. Another coumarin derivative, 6-acetyl-2,2-dimethylchroman-4-one, previously isolated from *A. princeps* shares similarities with DCC in structure and was shown to inhibit adipogenic differentiation via similar pathways to further support the AMPK activating ability of DCC [13].

In conclusion, DCC inhibited the adipogenic differentiation and lipid accumulation of hBM-MSCs through activation of AMPK-mediated Wnt/β-catenin signaling in vitro. Future studies are expected to further examine its potential to be utilized as a natural bioactive agent against bone marrow adiposity.

## 4. Materials and Methods

### 4.1. Materials

The isolation of DCC from *A. princeps* and the chemical characterization of the DCC were carried out as reported [22]. The NMR data of DCC used for identification is given in Figure S1. The human bone marrow-derived mesenchymal stromal cell (hBM-MSC) line, and cell culture (Mesenchymal Stem Cell Growth Medium, C-28009) and differentiation medium (Mesenchymal Stem Cell Adipogenic Differentiation Medium 2, C-28016) were purchased from PromoCell (Heildelberg, Germany). Other reagents used in cell culture, maintenance and differentiation including phosphate buffer saline (PBS) were purchased from Gibco BRL (New York City, NY, USA) unless otherwise noted. 3-[4,5-dimethylthiazol-2-yl]-2,5-diphenyltetrazolium bromide (MTT), dimethyl sulfoxide (DMSO), and isopropanol were purchased from Sigma-Aldrich (St. Louis, MO, USA). The AccuPrep Universal RNA extraction kit was purchased from Bioneer (Daejeon, Korea). The Cell Script All-in-One cDNA synthesis premix was obtained from CellSafe (Yongin, Korea). The NE-PER nuclear protein extraction kit, BCA protein assay kit for total protein quantification, and primary antibody against JNK were from Thermo Fisher Scientific (Waltham, MA, USA). Polyvinylidene fluoride membrane and enhanced chemiluminescence kit for the protein band detection were from Amersham Biosciences (Amersham, England, UK). Primary antibodies against PPARγ (#2443), CCAAT/enhancer-binding protein (C/EBP) α (#2295), p38 (#8690), phospho(p)-p38 (#4511), ERK (#4695), p-ERK (#4370), AMPK (#2603), and p-AMPK (#2531) were obtained from Cell Signal Technology (Danvers, MA, USA) while sterol regulatory element-binding protein 1c (SREBP1c) antibody (ab3259) was from Abcam (Cambridge, England, UK). Other primary secondary antibodies for Western blotting were purchased from Santa Cruz Biotechnology (Santa Cruz, CA, USA). For the immunocytochemical fluorescence staining, Alexa Fluor 488-conjugated secondary antibody (A-11008) was from Invitrogen (Waltham, MA, USA) while perilipin-1 (ab3526) and PPARγ (ab9256) primary antibodies were from Abcam. Solutions, buffers, and reagents (#12727) for the fluorescence staining were purchased from Cell Signal Technology.

### 4.2. Cell Culture and Differentiation

The hBM-MSCs were differentiated into adipocytes as previously reported [14]. Briefly, hBM-MSCs were grown to confluence, and the culture medium was swapped with differentiation medium two days later (day 0). The differentiation medium was changed with a fresh one every third day until most of the cells exhibited mature adipocyte characteristics by accumulating lipid droplets on the twelfth day of differentiation (day 12). DCC was introduced to differentiating cells on day 0 until the next medium change (day 3). One group of differentiated cells were not treated with DCC as a control group while another group of cells were not induced to differentiate (kept with growth medium) nor treated with DCC as a blank group.

### 4.3. Assessment of Cytotoxicity

The cytotoxic concentrations of DCC were assessed by traditional MTT assay. The hBM-MSCs were grown in 96-well plates to approximately 80% confluency. Next, cells were treated with increasing concentrations of DCC in growth medium and incubated for 72 h. After incubation, growth medium was replaced with a fresh one containing 20 μL MTT (1 mg/mL) instead of DCC, and the cells were kept at 37 °C in a 5% CO_2_ incubator for 4 h. The wells were then aspirated and supplied with 100% DMSO. The absorbance values of the wells at 540 nm were measured on a MultiSkan GO microplate reader (Thermo Fisher Scientific). The cytotoxicity of DCC was measured as a relative percentage of cell viability compared to the untreated group.

### 4.4. Lipid Droplet Staining

The Oil Red O staining was employed to demonstrate the cellular lipid accumulation as a marker of mature adipocyte. On day 12, the differentiated hBM-MSCs were washed with PBS and fixed on 6-well plates by addition on 10% formaldehyde for 1 h. After 1 h, the wells were aspirated and supplied with 0.5% Oil Red O staining solution (*wt*/*v*, dissolved in 3:2 isopropanol and distilled water). Staining was carried out by keeping the cells at room temperature for 1 h, after which the wells were aspirated again, washed with PBS and photographed using a microscope. Quantification of the Oil Red O stain bound by cellular lipid droplets was carried out via elution of the stain from cells through addition of 100% isopropanol. The wells were then measured for their absorbance at 500 nm on a MultiSkan GO microplate reader. The lipid accumulation was given as the stain absorbance percentage relative to the differentiated untreated control group.

### 4.5. Reverse Transcription-Polymerase Chain Reaction (RT-PCR)

The reverse transcription-polymerase chain reaction was performed using the total RNA isolated from the hBM-MSCs on day 12. Total RNA was extracted from cells with an AccuPrep Universal RNA extraction kit. The cDNA was synthesized from equal amounts of total RNA (2 μg) from each well. Reverse transcription was performed through the application of Cell Script All-in-One cDNA synthesis kit using a T100 thermocycler (Bio-Rad, Hercules, USA) with 42 °C for 60 min and 72 °C for 5 min settings. This was followed by standard PCR analysis consisting of 30 cycles of denaturing at 95 °C for 45 s, annealing at 60 °C for 1 min and extending at 72 °C for 45 s. The PCR reaction was carried out via Lune Universal PCR mix and following its instructions. The primers for the PCR analysis used were given in a previous report [14]. Finally, the PCR bands of the specific proteins were obtained after electrophoresing on 2% agarose gel and imaging with CAS-400SM imaging system (Davinch-K, Seoul, Korea).

### 4.6. Western Blot Analysis

The Western blot was carried out to detect the protein levels of specific proteins in hBM-MSCs on day 12. The homogenized cellular total protein lysates were obtained using RIPA buffer and vigorous pipetting. The lysates were then centrifuged at 4 °C for 15 min (13,000× *g*), and the supernatants were used for Western blot. Nuclear proteins were extracted from total cell lysates using a NE-PER nuclear extraction kit. The protein amounts of each well were assessed by BCA assay kit, and equal amounts of protein (20 μg) were loaded onto sodium dodecyl sulfate–polyacrylamide gel (4% stacking and 10% separating gels) for electrophoretic separation of the proteins. Next, the proteins were transferred to polyvinylidene fluoride membranes, which were then blocked with 5% skim milk in TBS-T buffer (*v*/*v*) for 1 h at room temperature. Blocked membranes were kept overnight at 4 °C with primary antibodies. Membranes were washed and hybridized with horseradish peroxidase-conjugated secondary antibodies. The hybridized protein bands were then visualized using enhanced chemiluminescence kit with a CAS-400SM imaging system.

### 4.7. Fluorescence STAINING

The cellular perilipin-1, PPARγ, and Wnt 10b proteins were visualized by immunofluorescence staining in hBM-MSCs on day 12. The hBM-MSCs were treated with the fixation, washing, and permeabilization reagents of the Immunofluorescence Application Solutions Kit according to its protocol. Next, cells were introduced to perilipin-1, PPARγ and Wnt10b primary antibodies. The cells were stained with Alexa Fluor 488-conjugated anti-rabbit secondary antibodies for visualization. For normalization, viable cells were also stained with DAPI (#8961; Cell Signaling Technology) containing ProLong Gold Antifade reagent. Stained cell images were taken using an Olympus fluorescence microscope equipped with a digital camera, and the images were then processed with the Multi Gauge software.

### 4.8. Statistical Analysis

The numerical data are given as mean of three separated results ± SD (*n* = 3). The statistically significant difference between two groups was decided according to the results of one-way analysis of variance followed by Duncan’s multiple range post hoc test. SAS (v9.1) software was used for statistical analysis and calculation of *p*-values. The meaningful significance was determined at *p* < 0.05.

## Data Availability

The data presented in this study are available on request from the corresponding author.

## Supplementary Materials

The following supporting information can be downloaded at: https://www.mdpi.com/article/10.3390/ijms23020653/s1.
