# Supplementary material for: Dracunculin Inhibits Adipogenesis in Human Bone Marrow-Derived Mesenchymal Stromal Cells by Activating AMPK and Wnt/β-Catenin Signaling
Source: Int J Mol Sci. 2022 Jan 7;23(2):653. doi: 10.3390/ijms23020653 (PMC8776130; doi:10.3390/ijms23020653)
Supplement: Supplementary file 1 [file ijms-23-00653-s001.zip › ijms-1522146-supplementary.pdf]

Dracunculin = 8-Methoxy-6,7-methylenedioxycoumarin

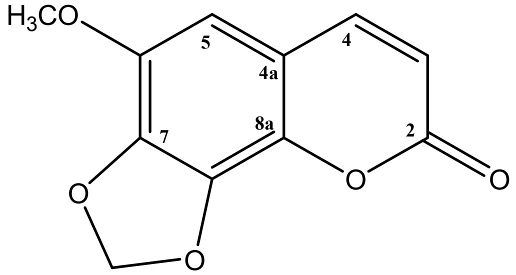

^{1}H NMR ($CDCl_3$, 300 MHz) δ 7.56 (1H, d, J = 7.6 Hz, H-4), 6.57 (1H, s, H-5), 6.26 (1H, d, J =9.6 Hz, H-3), 3.92 (3H, s, OMe); ^{13}C NMR ($CDCl_3$, 75 MHz) δ 159.7 (C, C-2), 143.5 (CH, C-4), 141.8 (C, C-6), 139.8 (C, C-8a), 135.0 (C, C-8), 133.8 (C, C-7), 114.3 (C, C-4a), 114.2 (CH, C-3), 105.0 (CH, C-5), 103.5 (CH_2, -OCH_2O-), 56.8 (CH_3, -OMe).

References for NMR data comparison

1) R. D. H. Murray, and M. Stefanovic, J. Nat. Prod., 1986, 49(3), 550-551.

2) J. H. Kwak, W. Y. Jang, O. P. Zee, and K. R. Lee, Planta Med, 1997, 63, 474-476.

3) M.-U. Rashid, S. Ali, M. Alamzeb, J. Igoli, C. Clements, S. Q. Shah, V. A. Ferro, A. I. Gray, and M. R. Khan, Pharm. Biol., 2014, 52(8), 983 - 987.
